# Variable Characteristics of Bacteriocin-Producing *Streptococcus salivarius* Strains Isolated from Malaysian Subjects

**DOI:** 10.1371/journal.pone.0100541

**Published:** 2014-06-18

**Authors:** Abdelahhad Barbour, Koshy Philip

**Affiliations:** Institute of Biological Sciences, Microbiology Division, Faculty of Science, University of Malaya, Kuala Lumpur, Malaysia; Teagasc Food Research Centre, Ireland

## Abstract

**Background:**

Salivaricins are bacteriocins produced by *Streptococcus salivarius*, some strains of which can have significant probiotic effects. *S. salivarius* strains were isolated from Malaysian subjects showing variable antimicrobial activity, metabolic profile, antibiotic susceptibility and lantibiotic production.

**Methodology/Principal Findings:**

In this study we report new *S. salivarius* strains isolated from Malaysian subjects with potential as probiotics. Safety assessment of these strains included their antibiotic susceptibility and metabolic profiles. Genome sequencing using Illumina’s MiSeq system was performed for both strains NU10 and YU10 and demonstrating the absence of any known streptococcal virulence determinants indicating that these strains are safe for subsequent use as probiotics. Strain NU10 was found to harbour genes encoding salivaricins A and 9 while strain YU10 was shown to harbour genes encoding salivaricins A3, G32, streptin and slnA1 lantibiotic-like protein. Strain GT2 was shown to harbour genes encoding a large non-lantibiotic bacteriocin (salivaricin-MPS). A new medium for maximum biomass production buffered with 2-(N-morpholino)ethanesulfonic acid (MES) was developed and showed better biomass accumulation compared with other commercial media. Furthermore, we extracted and purified salivaricin 9 (by strain NU10) and salivaricin G32 (by strain YU10) from *S. salivarius* cells grown aerobically in this medium. In addition to bacteriocin production, *S. salivarius* strains produced levan-sucrase which was detected by a specific ESI-LC-MS/MS method which indicates additional health benefits from the developed strains.

**Conclusion:**

The current study established the bacteriocin, levan-sucrase production and basic safety features of *S. salivarius* strains isolated from healthy Malaysian subjects demonstrating their potential for use as probiotics. A new bacteriocin-production medium was developed with potential scale up application for pharmaceuticals and probiotics from *S. salivarius* generating different lantibiotics. This is relevant for the clinical management of oral cavity and upper respiratory tract in the human population.

## Introduction

Bacteriocin or bacteriocin-like inhibitory substances (BLIS) are peptide molecules produced by Gram-positive bacteria and some genera of Gram negative bacteria [1]–[3]. Lactic acid bacteria are generally considered to be non-pathogenic (with some exceptions such as *Streptococcus mutans* which causes dental caries) and can produce different kinds of bacteriocins such as nisin produced by *Lactococcus lactis* [4]–[7], plantaricins produced by *Lactobacillus plantarum* [8]–[10], mutacins produced by *Streptococcus mutans* [11]–[15] and salivaricins produced by *Streptococcus salivarius* [16]–[20]. *S. salivarius* is a species of lactic acid bacteria colonizing the human oral cavity [21].

Some strains of *S. salivarius* such as strains K12 and M18 are now being used as probiotics worldwide due to their capability to produce different kinds of bacteriocins called lantibiotics [18], [22], [23]. Lantibiotics are heat stable ribosomally synthesized small molecules produced by some strains of gram positive bacteria with therapeutic potential in treating infectious diseases [24]–[29].

To compete better in the oral ecosystem, *S. salivarius* produce different kinds of lantibiotics such as salivaricin A, salivaricin B, salivaricin 9 and salivaricin G32 [16]–[18], [20]. It has been noticed that bacteriocin or BLIS molecules are not the only useful metabolites produced by *S. salivarius*. Levan-sucrase is one of the important molecules secreted by *S. salivarius* [30]. Levan-sucrase or fructosyltransferase (FTF) attack the fructose moiety of sucrose and polymerize it into fructans which possess levan structure. Levan is a homo-polysaccharide, non-mutagenic, non-toxic, soluble dietary fiber with significant prebiotic effects through stimulating the growth and activity of selected probiotic bacteria in the colon which can improve the host’s health [31]. Levan may also contribute to human health by its antitumor [32], [33] and antidiabetic activities [34]. Microbial levan is of great importance for its variable applications in food, cosmetics and pharmaceutical industries [35]. Levan can also be used in drug delivery formulations as a coating material and carrier for fragrances and flavors [36], [37].

In this study we describe the different characteristics of strains of *S. salivarius* isolated from healthy Malaysian subjects which showed antagonism against selected Gram-positive bacteria. The preliminary safety assessment study of these strains did not detect any streptococcal virulence genes and demonstrated the susceptibility of the *S. salivarius* strains to a number of classes of antibiotics. The stability of the metabolic profile was also investigated in this study and showed some variations among the strains.

A novel method was developed for direct detection of the levan-sucrase enzyme in crude extracts of *S. salivarius* cells using LC/MS-MS technology. The *de novo* amino acid sequence of the enzyme showed similarity to that produced by other lantibiotic producing *S. salivarius* strains. However, genome sequencing of strain YU10 helped to fully characterise the gene encoding levan-sucrase production. Due to the high level of levan-sucrase production and the secretion of lantibiotics, strains presented in this study can play a great role in pharmaceutical applications as a source of bacteriocins that can be used as probiotics and/or prebiotics to improve human oral health. This study also led to the development of a new medium to obtain higher biomass levels of *S. salivarius* and lantibiotic production during aerobic fermentation when compared with other commercial media. This new medium can be used to enhance bacteriocin production by *S. salivarius* which may help to develop new oral probiotics.

## Results

### 1. Antagonism Activity of Bacteriocin Producing *S. salivarius*

To determine which medium can be used to recover the highest levels of lantibiotics or bacteriocins, the deferred antagonism assay was applied using different solid media as a production system. Strain K12 (salivaricins A and B producer) gave the broadest antagonism spectrum against a number of selective indicators as shown in Table 1. BACa and TYECa appeared to be the best media for lantibiotics production with strain K12. When PTNYSMES medium was used, strain K12 failed to inhibit the growth of *Lactobacillus delbrueckii* subsp. *bulgaricus*. Strains YU10 and NU10 inhibited most of the streptococcal strains used in this study (but not *Streptococcus mutans*) while the levels of lantibiotics secreted by these strains were improved when PTNYSMES medium was used as the production medium. The inhibitory spectrum of both NU10 and YU10 included one *Listeria monocytogenes* strain (partial inhibition). When blood was used as a supplementary component in the production medium (BACa), strain GT2 expressed *S. pyogenes*-inhibitory activity. Surprisingly, no anti-*Micrococcus luteus* inhibitory activity was detected when strain GT2 was grown onto BACa plates indicating that the bacteriocin produced may not be a lantibiotic since *M. luteus* is known for its extreme susceptibility to lantibiotics. However, when GT2 was grown on PTNYSMES and TSYECa media, there was some inhibition towards *M. luteus*. In all media used with strain GT2 as a producer, most of the inhibitory activity was eliminated when the media were heated at 70°C for 30 minutes (data not shown). This finding indicates that strain GT2 may express heat labile bacteriocin. It was noticed that strain YU10 does not exhibit self-immunity as it showed significant antagonism activity towards itself when tested for self-immunity assay with all kinds of media. However, when PTNYSMES was used in this test, most of the producers showed lack of self-immunity (Table 1).

**Table 1:** Deferred antagonism assay of different BLIS producing *S. salivarius* strains using different production media.

Indicator microorganisms	Antagonism activity of *S. salivarius* isolates towards indicator microorganisms using different production media																			
	NU10					YU10					GT2					K12				
	1	2	3	4	5	1	2	3	4	5	1	2	3	4	5	1	2	3	4	5
*A. naeslundii* TG2	−	−	−	−	−	−	−	−	−	−	−	−	−	−	−	−	−	−	−	−
*B. cereus* ATCC14579	−	−	−	−	−	−	−	−	−	−	−	−	−	−	−	−	−	−	−	−
*Corynebacterium. spp* GH17	+++	+++	+++	−	++	++	+++	+++	−	++	++	−	+++	−	−	+++	+++	+++	−	+++
*E. faecium* C1	(+)^H^	(+)^H^	−	−	(+)^H^	−	−	−	−	−	−	−	−	−	−	++	++	++	−	++
*H. parainfluenzae* TONEJ11	−	−	−	−	−	−	−	−	−	−	−	−	−	−	−	(+)^H^	−	−	−	−
*L. bulgaricus* M8	−	−	−	−	−	−	−	(+)^H^	−	−	−	−	(+)^H^	−	−	++	+	−	−	+
*L. lactis* ATCC11454	(+)^H^	−	−	−	−	(+)^H^	−	−	−	−	−	−	−	−	−	+++	+++	++	−	++
*L. monocytogenes* NCTC	+	++	++	−	++	−	−	(+)^H^	−	−	−	−	−	−	−	+	+	+	−	++
*M. luteus* ATCC10240	+++	+++	+++	−	+++	++	+++	+++	−	+++	++	−	+++	−	+	+++	+++	+++	−	+++
*S. aureus* RF122	−	−	+	−	−	−	−	−	−	−	−	−	−	−	−	++	++	+	−	(+)^H^
*S. equisimilis* ATCC12388	++	+++	+++	−	++	+	+++	+++	−	+++	++	+++	+	−	−	+++	+++	+++	−	+++
*S. gordonii* ST2	−	−	−	−	−	−	−	−	−	−	−	−	−	−	−	−	−	−	−	−
*S. mutans* GEJ11	−	−	−	−	−	−	−	−	−	−	−	−	−	−	−	++	++	++	−	++
*S. pyogenes*ATCC12344	++	+++	+++	−	+++	+	+++	+++	−	+++	+	+++	++	+	−	+++	+++	+++	−	+++
*S. pyogenes* ATCC12348	++	+++	+++	−	+++	+	+++	+++	−	+++	+	+++	++	−	−	+++	+++	+++	−	+++
*S. salivarius* GT2	+	++	++	−	+	+	++	+	−	−	−	−	+	−	−	++	++	+	−	++
*S. salivarius* K12	−	−	+	−	−	−	−	+	−	−	−	−	+	−	−	+	−	+	−	−
*S. salivarius* NU10	−	−	−	−	−	−	−	+	−	−	+	−	+	−	−	+	+	+	−	+
*S. salivarius* YU10	+	+	++	−	−	(+)^H^	(+)^H^	++	−	(+)^H^	−	−	++	−	−	++	+++	+	−	++
*S. sanguinis* ATCC10556	−	(+)^H^	+++	−	(+)^H^	−	(+)^H^	+++	−	(+)^H^	−	(+)^H^	+++	−	−	+++	++	+++	−	(+)^H^
*W. confusa* A3	−	−	−	−	−	−	−	−	−	−	−	−	−	−	−	+	+	+	−	+

+Inhibition zone <0.75 cm, ++inhibition zone = 0.75–1 cm, +++inhibition zone >1 cm, −no inhibition.

H Hazy zone of inhibition.

1 TSYECa: Tryptic Soy agar supplemented with 2% Yeast extract and 0.1% CaCO3.

2 BACa: Columbia agar base supplemented with 5% whole defibrinated sheep blood and 0.1% CaCO3.

3 PTNYSMES medium supplemented with 1.5% bacteriological agar.

4 CAB+NBCSCa: Columbia agar base supplemented with 5% New born calf serum and 0.1% CaCO3.

5 M17 agar supplemented with 2% Yeast extract, 1% Sucrose and 0.1% CaCO3.

### 2. Genes Encoding Salivaricins Production

Strain K12 is known to harbour the structural genes *salA*, *sboA* and *MPS var* encoding salivaricins A, B and MPS variant production respectively while no *sivA*, *MPS* or *slnA* were detected within this strain. Strains NU10 and YU10 were shown previously to harbour *sivA* (structural gene for salivaricin 9 production) and *salA* [38]. In this study, strain YU10 was found to harbour structural genes encoding for streptin, salivaricin A3 and salivaricin G32 lantibiotics and the slnA1 lantibiotic-like protein. However, salivaricin G32 was the only bioactive lantibiotic expressed and detected in strain YU10 and it seems that not all of these genes are likely to be expressed. Genome sequencing of strain YU10 confirmed the presence of genes mentioned above. It was found that salivaricin A is quite widely distributed in streptococcal species but the locus is often quite defective and so the inhibitory product is not expressed even though the salivaricin A immunity component may be functional. Strain GT2 was shown to harbour *MPS* and *MPS var* genes encoding the production of salivaricin MPS and MPS variant respectively.

### 3. Metabolic Profiles and Biochemical Characteristics of *S. salivarius* Isolates

The stability of the metabolic profiles of *S. salivarius* strains was carried out using API kits. The biochemical characteristics of each strain provided important information on the needs and criteria of each isolate to achieve optimal growth. YU10 was the only strain tested in this study that showed D-sorbitol positive reaction. This information can also be used as a differentiation method for YU10 detection. Unlike NU10 and YU10, strains K12 and GT2 are able to use inulin or galactose as a carbon source. NU10 was the only strain with positive reaction for amygdalin while GT2 was the only strain that fermented melibiose. Strain K12 also differed with the other strains by fermenting D-tagatose. NU10 was the only strain that failed to ferment lactose and this is unusual for lactic acid bacteria. However, when lactose was used as a carbon source during media development, strain NU10 was unable to grow adequately. YU10 was the only strain with trehalose negative reaction (Table 2).

**Table 2:** API 20 STREP and API 50*S. salivarius* isolates.

API 20 STREP	K12	NU10	NU10Ω	YU10	YU10Ω	GT2	GT2Ω
Acetoin production	+	+	+	+	+	+	+
Hydrolysis (HIP puric acid)	−	−	−	−	−	−	−
B-Glucosidase hydrolysis (Esculin)	+	+	+	+	+	+	+
Pyrrolidonyl arylamidase	−	−	−	−	−	−	−
α-Galactosidase	−	−	−	−	−	−	+
β-Glucuronidase	−	−	−	−	−	−	−
β-Galactosidase	−	−	−	−	−	−	−
Alkaline phosphatase	+	+	−	+	+	+	+
Leucine aminopeptidase	+	+	+	+	+	+	+
Arginine dihydrolase	−	−	−	−	−	−	−
D-Ribose	−	−	−	−	−	−	−
L-Arabinose	−	−	−	−	−	−	−
D-Mannitol	−	−	−	−	−	−	−
D-Sorbitol	−	−	−	+	+	−	−
D-Lactose	+	−	−	+	+	−	−
D-Trehalose	+	+	+	−	−	+	+
Inulin	+	−	−	−	−	-	+
D-Raffinose	+	+	+	+	+	+	+
starch	+	+	+	+	+	+	+
glycogen	−	−	−	−	−	−	−
**API 50 CHL**	**K12**	**NU10**	**NU10** Ω	**YU10**	**YU10** Ω	**GT2**	**GT2** Ω
L-Arabinose	−	−	−	−	−	+	+
Ribose	−	−	−	−	−	+	+
Galactose	+	−	−	−	−	+	+
D-Glucose	+	+	+	+	+	+	+
D-Fructose	+	+	+	+	+	+	+
D-Mannose	+	+	+	+	+	+	+
Sorbitol	−	−	−	+	+	−	−
a-Methyl-D-glucoside	−	−	−	−	−	−	−
N-Acetyl glucosamine	+	+	+	+	+	−	−
Amygdalin	−	+	+	−	−	−	−
Arbutin	+	+	+	+	+	+	+
Esculin	+	+	+	+	+	+	+
Salicin	+	+	+	+	+	+	+
Cellobiose	+	+	+	+	+	+	+
Maltose	+	+	+	+	+	+	+
Lactose	+	−	−	+	+	+	+
Melibiose	−	−	−	−	−	+	+
Saccharose	+	+	+	+	+	+	+
Trehalose	+	+	+	−	−	+	+
Inulin	+	+	+	+	+	+	+
Melezitose	−	−	−	−	−	−	−
D-Raffinose	+	+	+	+	+	+	+
β-Gentiobiose	−	+	+	+	+	+	+
D-Tagatose	+	−	−	−	−	−	−

Ω Strain was sub-cultured subsequently for 20 times before testing for metabolic profile.

### 4. Antibiotics Susceptibility

*S. salivarius* isolates tested in this study were assessed to be moderately resistant to gentamicin and this finding is similar to what was published for strain K12 and other *S. salivarius* isolates [39]. According to CLSI breakpoints for ofloxacin, all *S. salivarius* strains tested in this study were sensitive to this antibiotic (inhibition zone >16 mm). Furthermore, *S. salivarius* strains tested in this study were sensitive to several routinely used antibiotics for the control of upper respiratory tract infections. It was noticed that strain YU10 showed intermediate susceptibility levels to erythromycin with 19.7 mm zone of inhibition. However, the other strains NU10, GT2 and K12 were susceptible to the same antibiotic. No significant differences regarding antibiotic susceptibility were observed after two years of storage indicating that the strains tested in this study are reasonably stable. Full results of antibiogram are listed in Table 3.

**Table 3:** Antibiotic disc sensitivities of *S. salivarius* isolates.

Antibiotic (discs)	Disc content	Inhibition zone size (mm) for *S. salivarius* strains							CLSI Breakpoints^a^		
		NU10§	NU10¥	YU10§	YU10¥	GT2§	GT2¥	K12	S	I	R
Penicillin G	10 µg	21	23	22	22	20	20	21.5	–	–	–
Penicillin V	10 µg	30.5	30	33.3	30.5	27	28	32	–	–	–
Ampicillin	10 µg	26	27	26	27	24	23	28.5	–	–	–
Amoxycillin	10 µg	30	32	31.5	29.5	25.6	27	30	–	–	–
Erythromycin	15 µg	30.5	30.2	19.7	19.7	32	29.5	30	≥21	16–20	≤15
Tetracyclin	30 µg	31	31	31.5	31.5	31.7	32	29	≥23	19–22	≤18
Gentamycin	10 µg	14.5	14.5	19	18	16	15.5	13.5	–	–	–
Clindamycin	2 µg	28.5	29	31.7	31	27	28	29	≥19	16–18	≤15
Ofloxacin	5 µg	21	21	20.5	20.5	20	19.5	18.3	≥16	13–15	≤12
Streptomycin	10 µg	12	11	13	13.5	10	11	9.5	–	–	–
Novobiocin	5 µg	13.5	14	13.5	12	13	13	12.5	–	–	–
Vancomycin	30 µg	20	20	24	22.5	22	21.5	21	≥17	–	–
Cloramphenicol	30 µg	26	25	26.5	26	27.5	25.5	27	≥21	18–20	≤17

a S: susceptible, I: intermediate, R: resistant.

¥ Original isolate.

§ Routinely subcultured isolate.

### 5. Genome Sequencing of Strains NU10 and YU10

**Figure 1:**
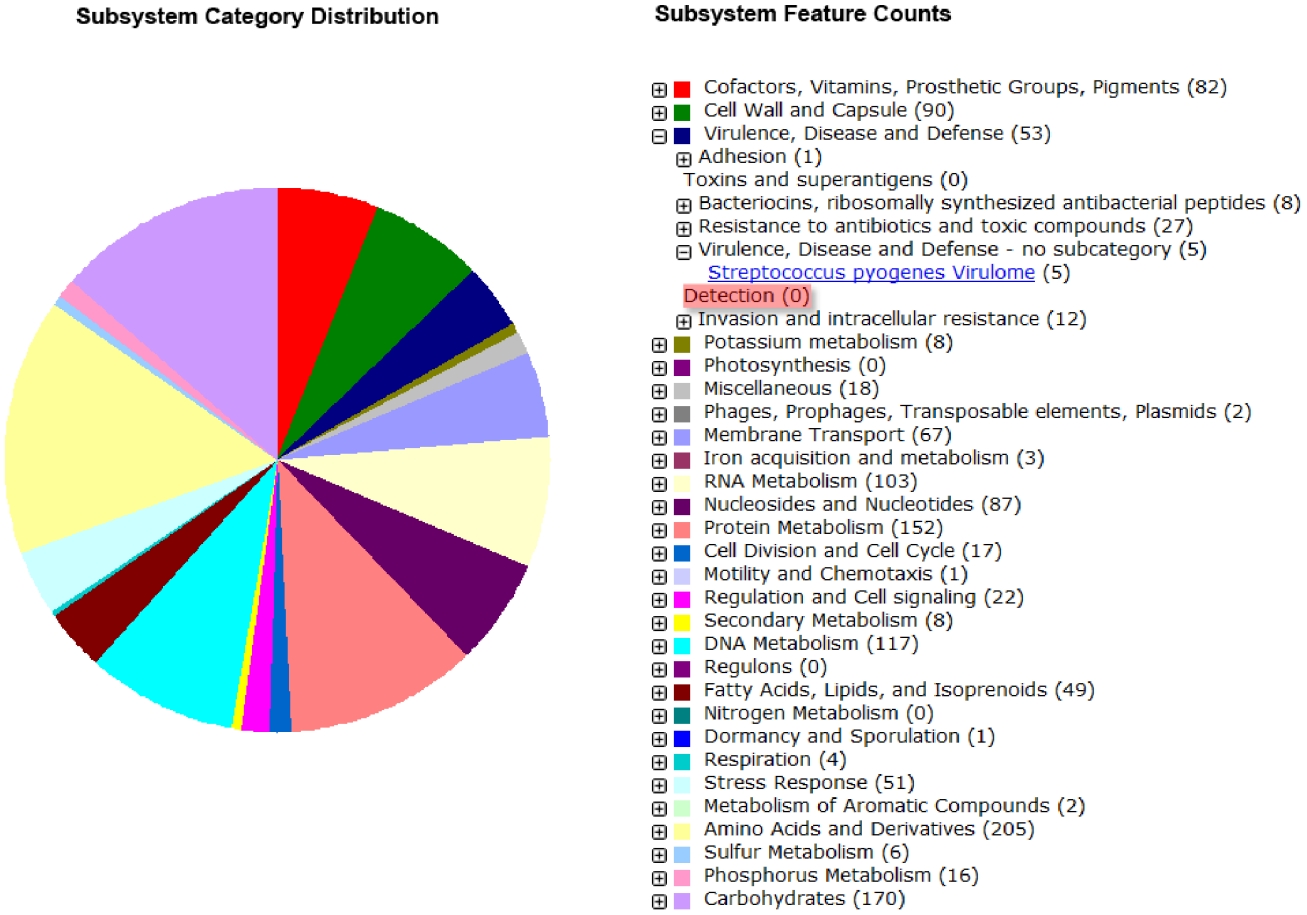
Subsystem feature counts of *S. salivarius* strains NU10 and YU10 detected by RAST. No *S. pyogenes* virulence determinants were detected.

Genome sequencing of strains NU10 and YU10 was performed with Illumina Miseq sequencer. Total of 2,344,494 and 2,345,259 pair-end reads were generated with contigs numbering 51 and 48 for NU10 and YU10 respectively. Genome annotation was performed using RAST [40]. A total of 2146 coding sequences (CDSs) and 38 structural RNAs were predicted in strain NU10 while 2161 CDSs and 41 structural RNAs were predicted in strain YU10. Genes encoding for streptin, salivaricin A3, salivaricin G32 lantibiotics and slnA 1 lantibiotic-like protein were detected in the genome draft of strain YU10. However, only salivaricin A3, salivaricin G32 and slnA 1 lantibiotics showed 100% homology to genes present in strain YU10. The gene encoding for streptin present in YU10 genome has only 82% homology to streptin due to some mutations (Text S1). Furthermore, strains YU10 and NU10 were shown to be free of any streptococcal pyrogenic exotoxins, streptococcal superantigen A (SSA), streptococcal mitogenic exotoxin Z (SmeZ) and streptodomase B. Other streptococcal virulence factors listed in Table 4 were also investigated and none of them were present in YU10 or NU10 genomes. Figure 1 shows subsystem feature counts of *S. salivarius* strains NU10 and YU10 detected by RAST. Under virulence, disease and defense category we can clearly observe that no *Streptococcus pyogenes* virulence determinants, toxins or superantigens were detected. Other factors related to bacteriocin production, protein synthesis and adhesion factors are not part of the toxin and virulence factors. This assessment suggests that both YU10 and NU10 are safe for future use as probiotics.

**Table 4:** Virulence assessment for *S. salivarius* strains YU10 and NU10.

Virulence determinant	Gene designation	*S. salivarius* strains	
		YU10	NU10
M-protein	*emm*	–	–
Protein H	*sph*	–	–
Streptokinase	*Ska*	–	–
CAMP factor	*cfa*	–	–
Streptolysin S	*SagA*	–	–
Streptolysin O	*slo*	–	–
Hyaluronate lyase	*hyl*	–	–
Nicotin adenine dinuclutide glycohydrolase	*nga*	–	–
Streptococcal pyrogenic exotoxin A	*SpeA*	–	–
Streptococcal pyrogenic exotoxin B	*SpeB*	–	–
Streptococcal pyrogenic exotoxin C	*SpeC*	–	–
Streptococcal pyrogenic exotoxin G	*SpeG*	–	–
Streptococcal pyrogenic exotoxin H	*SpeH*	–	–
Streptococcal pyrogenic exotoxin I	*SpeI*	–	–
Streptococcal pyrogenic exotoxin J	*SpeJ*	–	–
Streptococcal pyrogenic exotoxin K	*SpeK*	–	–
Streptococcal pyrogenic exotoxin L	*SpeL*	–	–
Streptococcal pyrogenic exotoxin M	*SpeM*	–	–
Streptococcal superantigen A	*SSA*	–	–
Streptococcal metogenic exotoxin Z	*SmeZ*	–	–
Streptodornase B	*SdaB*	–	–
Fibrinogen binding protein	*fba*	–	–
Fibrotectin-binding protein (protein F)	*prtF*	–	–
Protein G-related alpha 2 macroglobulin binding protein	*grab*	–	–
Streptococcal inhibitor of complement	*SIC*	–	–
Immunoglobulin G-endopeptidase	*IdeS*	–	–
Secreted endo-β-N-acetylglucosaminidase	*ndoS*	–	–
C5a peptidase	*ScpA*	–	–
Fibronectin-binding protein	*FBP*	–	–
Serum opacity factor	*SOF*	–	–
C3 family ADP-ribosyltransferase	*SpyA*	–	–
Serine endopeptidase	*ScpC*	–	–
Hyaluronan synthase	*HasA*	–	–
Collagen-like surface protein	*SclB*	–	–

(−): absence of the virulence factor.

### 6. Developing New Bacteriocin-production Medium

**Figure 2:**
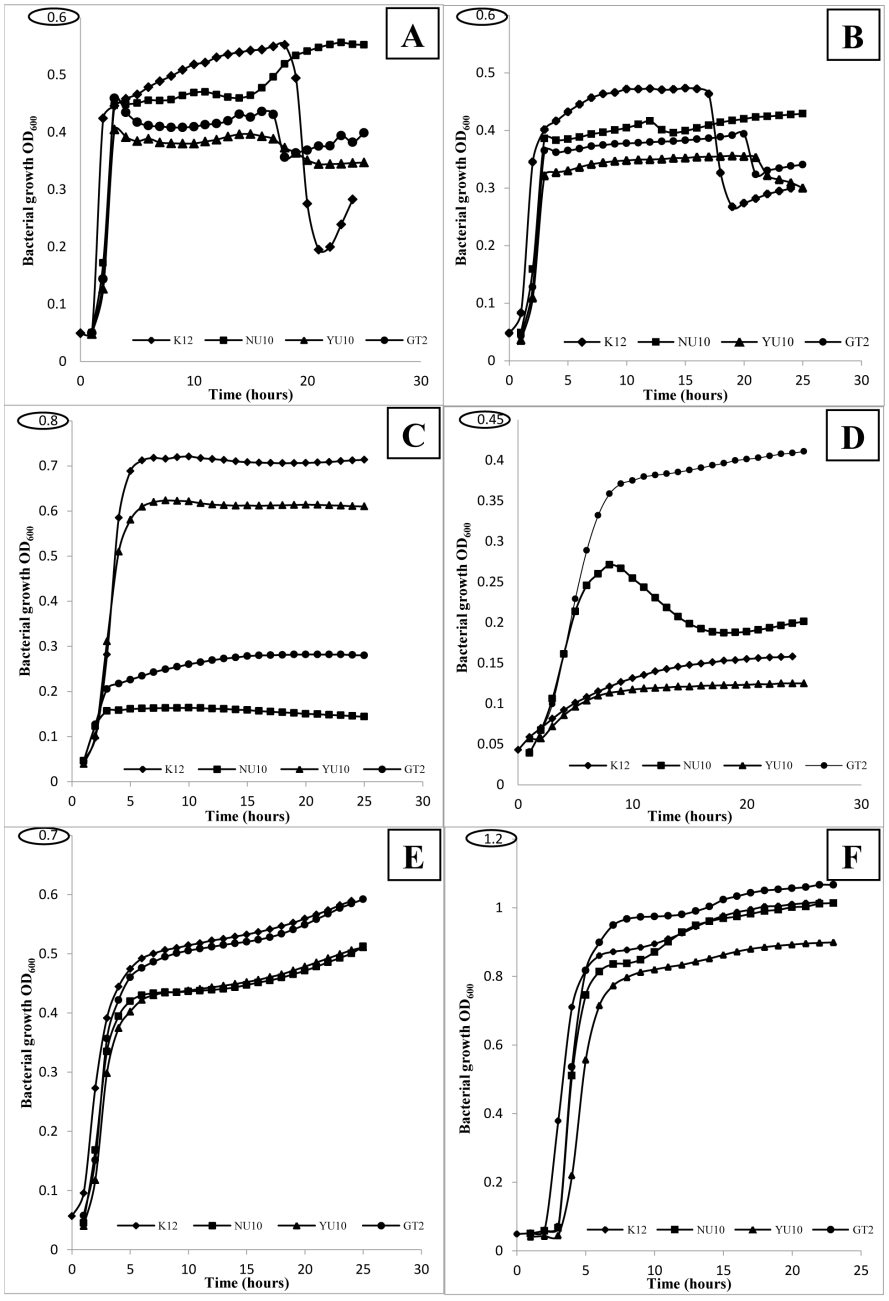
Growth kinetics of *S. salivarius* strains grown aerobically in different media. A: THB (BD), B: BHI (BD), C: M17 (Merck), D: MRS (Merck), E: YNS and F: PTNYSMES.

The newly developed medium used in this study helped to increase the biomass of *S. salivarius* cultures grown aerobically. The carbon source (sucrose) used within this medium was adequate to grow all the strains as some of them cannot process lactose (strain NU10). Usually, *S. salivarius* requires CO_2_ enriched atmosphere to grow adequately and thereby produce lantibiotic molecules. However, in this study we tried to develop enriched medium that helped to cultivate *S. salivarius* aerobically without any supplemented CO_2_. However, strain NU10 showed some susceptibility to levels of CO_2_ (3–5%) and it did not grow in M17 medium (Merck) which is supplemented originally with lactose as a carbon source. This finding confirms the metabolic profile of strain NU10 which was unable to uptake lactose. Strain GT2 also showed weak growth in M17 medium used in this study and this event cannot be linked to carbon source since this strain showed positive reaction for lactose test. When THB or BHI media were used, some lytic activities were observed after 20 hours of bacterial growth as the OD_600_ values started to decrease. Although it is designed for lactic acid bacteria, MRS medium failed to grow *S. salivarius*. However, strain GT2 showed better growth (but still weak, OD_600_ = 0.4) when grown in this medium as compared with other *S. salivarius* strains. YNS medium showed better bacterial growth compared to other commercial media especially for strains GT2 and K12. However, the newly developed PTNYSMES was the best medium tested for *S. salivarius* growth in this study and showed a significant increase in the optical density of all the isolates. Compositions of all media used are listed in Table 5. The differences in pH values before and after 22 hours of fermentation for each medium are listed in Table 6. All isolates reached the stationary phase of growth in just 10 hours and showed no autolytic activities even after 24 hours. OD_600_ = 1 was achieved with strains K12, NU10 and GT2 while strain YU10 also showed good biomass accumulation with OD_600_ = 0.9 (Figure 2).

**Table 5:** Typical compositions for media used to cultivate *S. salivarius.*

Typical Composition	Media used for *S. salivarius* growth						
	PTNYSMES	YNS	M17	MRS	BHI	THB	BruB¥
**Nitrogen source**							
Peptone	**✓**		**✓**	**✓**	**✓**		**✓**
Tryptone	**✓**						
Neopeptone	**✓**	**✓**				**✓**	
Yeast extract	**✓**	**✓**	**✓**	**✓**			**✓**
Meat extract			**✓**	**✓**			
Heart/Brain infusion					**✓**	**✓**	
Digest of animal tissue							**✓**
**Carbon source**							
Dextrose				**✓**	**✓**	**✓**	**✓**
Lactose			**✓**				
Sucrose	**✓**	**✓**					
**Vitamins**							
Ascorbic acids	**✓**		**✓**				
**Salts**							
Sodium chloride	**✓**				**✓**	**✓**	**✓**
Disodium phosphate					**✓**	**✓**	
Sodium carbonate							
Magnesium sulfate			**✓**	**✓**			
Manganese sulfate	**✓**			**✓**			
Sodium bisulfite				**✓**			**✓**
Sodium acetate	**✓**			**✓**			
Sodium carbonate						**✓**	
**Buffers**							
MES	**✓**						
di-potassium hydrogen phosphate				**✓**			
Na-β-Glycerophosphate			**✓**				

¥ Brucella broth (Difco).

**Table 6:** Variation of the pH values of *S. salivarius* cultures grown in different media after 22 hours of growth.

Medium	Initial pH of the medium/final pH of the culture after 22 h fermentation			
	K12	GT2	NU10	YU10
THB	7.8/5.00	7.8/5.08	7.8/5.16	7.8/5.23
BHI	7.01/5.16	7.01/5.38	7.01/5.33	7.01/5.64
M17	7.13/5.57	7.13/6.55	7.13/6.82	7.13/5.74
MRS	5.59/4.90	5.59/5.00	5.59/5.03	5.59/5.59
Brucella Broth	6.53/5.09	6.53/5.14	6.53/5.08	6.53/5.11
YNS	6.71/3.83	6.71/3.78	6.71/3.84	6.71/3.91
PTNYSMES	6.51/4.31	6.51/4.30	6.51/4.27	6.51/4.33

### 7. Salivaricin 9 and Salivaricin G32 Production

**Figure 3:**
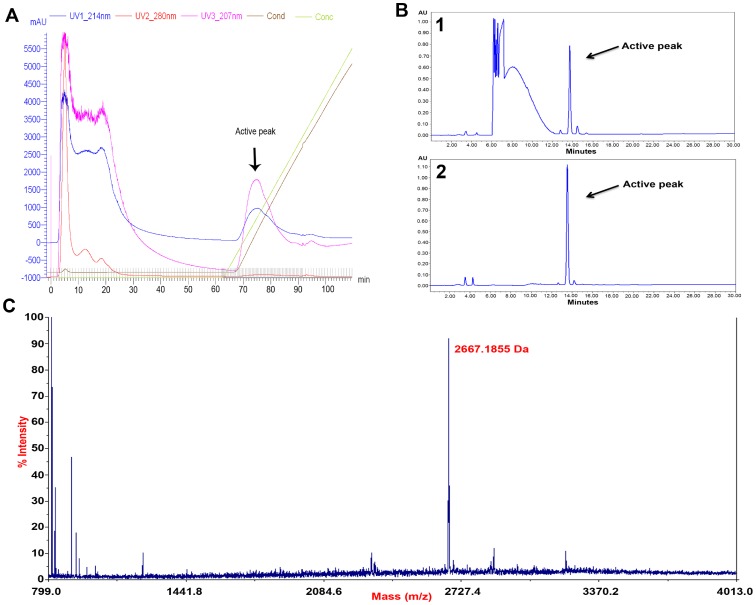
Purification and detection of salivaricin G32 produced by strain YU10 grown in PTNYSMES. A: Cation exchange chromatography of the cell extract using SP FF column, B1: RP HPLC of the pooled active fractions of salivaricin G32 obtained from A, B2: second RP HPLC of the active fraction obtained by B1. C: MALDI-TOF (MS) analysis of the pure salivaricin G32.

Attempts to recover lantibiotics from *S. salivarius* cells grown in PTNYSMES medium were successful. Both strains NU10 and YU10 were grown for 24 hours in this medium and the lantibiotics were subsequently recovered by cell extraction followed by further chromatography techniques for lantibiotic purification. MALDI-TOF (MS) analysis showed that like our previous report [38], salivaricin 9 (2560 Da) was produced by strain NU10 using PTNYSMES medium in the current study. Furthermore, salivaricin G32 (2667 Da) (Figure 3) was the only detectable and known lantibiotic produced by this strain when grown in the new medium. Salivaricin A was not produced or detected by strains YU10 or NU10 using this medium even though the strains harbour the structural gene encoding this lantibiotic. The production experiment was repeated without adjusting the pH of the medium after fermentation (without adsorption) to calculate levels of lantibiotics attached to the producer cells. However, attempts to recover lantibiotics from the cell-free supernatant of this preparation using 80% ammonium sulphate saturation as described previously [41] showed that 60–70% of lantibiotic produced by NU10, YU10 and K12 strains presented in this study is cell-wall associated peptide (Table 7). The bacteriocin units (arbitrary units) were calculated as mentioned previously [38].

**Table 7:** Inhibitory activity recovered from cell extracts and cell-free supernatants of *S. salivarius* cultures.

Inhibitory activity recovery (From 1 L culture)§	Lantibiotic-producing *S. salivarius* strains		
	NU10	YU10	K12
Total activity	4.8×10^4 ^AU (100%)	1.32×10^4 ^AU (100%)	1.02×10^5 ^AU (100%)
Cell extract activity	3.2×10^4 ^AU (66.6%)	9.6×10^3 ^AU (72.7%)	6.4×10^4 ^AU (62.5%)
Cell-free supernatant activity	1.6×10^4 ^AU (33.4%)	3.6×10^3^ AU (27.2%)	3.84×10^4^ AU (37.5%)

§ Strains were grown in PTNYSMES medium.

AU: arbitrary unit.

### 8. Levan-sucrase Detection and Characterization

**Figure 4:**
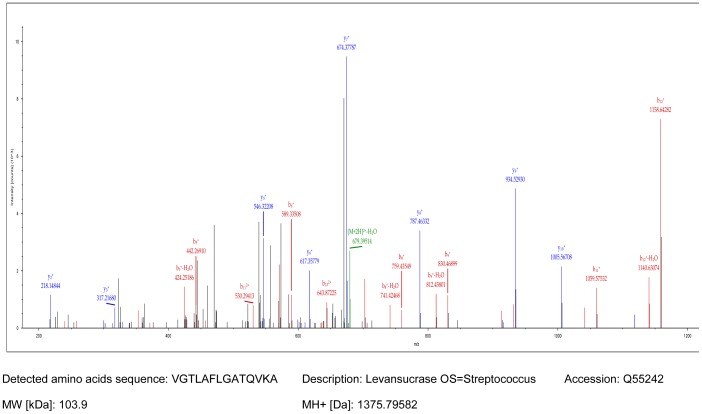
Detection of levan-sucrase enzyme produced by strain YU10 using ESI-LC-MS/MS analysis.

Cell-associated levan-sucrase was extracted from *S. salivarius* cells of strain YU10. Advanced LC-MS/MS method was developed for direct detection of this unique enzyme from the cell extract using reverse phase chromatography. The peptide which matched the levan-sucrase enzyme (accession: Q55242) contains 14 residues (VGTLAFLGATQVKA). The match was considered significant by the search algorithm with a score of 78.88 and coverage of 1.44. This defines matches with ion score of 51 for identity and charge of 2. Retention time for levan-sucrase was 38.71 minutes with MH+ [Da] = 1375.79582 (Figure 4). Genome sequencing of strain YU10 revealed the structural gene encoding for levan-sucrase or fructosyltransferase (FTF) production. Full characterization of the gene (*ftf*) with *in silico* protein translation is provided as support information (Text S2). The *ftf* region of strain YU10 was compared to *ftf* region in the commercial probiotic strain M18 genome [23] and both regions were almost identical. In addition to fructosyltransferase, this region included gene encoding for levanase production (Figure S1).

## Discussion

Three different salivaricin-producing *S. salivarius* strains isolated from Malaysian subjects were evaluated in this study and shown to produce different kinds of BLIS molecules some of which are lantibiotics (sal9 and salG32). Gene encoding a large peptide molecule salMPS (accession number: AGBV01000006) was also detected in one of the strains (GT2).

Strains K12, NU10 and YU10 produced inhibitory activity when grown on different media including M17 (Difco), BACa, PTNYMES and others mentioned in the results. On the other hand, strain GT2 failed to produce significant anti-*S. pyogenes* inhibitory activity when grown on media which was not supplemented with blood but produced significant inhibitory activity against *S. pyogenes* when grown on BACa. This indicates that the production of anti-*S. pyogenes* inhibitory activity by this strain is likely to be dependent on blood components. This characteristic is similar to salivaricin MPS-like peptide which is a large bacteriocin molecule [42]. Further analysis showed that strain GT2 harbours the structural gene encoding for salivaricins MPS and MPS variant productions.

Strain NU10 was shown to harbour structural genes encoding salivaricins A and 9 previously but only sal9 could be produced and detected as an active peptide in the present study. Strain YU10 was shown to harbour genes encoding salivaricins A3, G32, streptin and slnA1 lantibiotic-like protein, however, only salG32 was detected and recovered from this strain.

The strains in this study also showed some variations in their metabolic profiles. Surprisingly, strain NU10 showed a negative reaction for lactose fermentation and when the strain was propagated in growth medium containing lactose as the only carbon source, it showed significantly weaker growth and total absence of any lantibiotic production. A previous study that was done in our laboratory [38] showed that this strain is a producer of salivaricin 9. The maximum yield of BLIS activities was recovered when sucrose was used as the carbon source.

The use of commercial media including THB and BHI in aerobic condition resulted in a drop of OD_600_ reading that is apparently attributed to microbial cell lysis. The reason for this lysis in aerobic condition is still unknown and perhaps the aerobic condition is not ideal for strain K12 and other *S. salivarius* isolates when THB or BHI media are used for propagation (Figure 2). In the current study, a newly developed medium buffered with MES helped to enhance the biomass and bacteriocin production by *S. salivarius* which grew well in an aerobic atmosphere. This finding can solve the problem of scaling-up the culture in large scale bioreactors for probiotic and/or lantibiotic production. Previous study showed that buffering the medium with MES helped to achieve higher biomass levels of *Streptococcus thermophilus* [43]. Using organic buffers for bacteriocin production helps to prevent extreme drop in pH of medium due to the production of lactic acid or other substances.

It has been noticed that 60–70% of the bacteriocins recovered in this study were cell-wall associated peptides bound to the producer cells while the rest of the inhibitory peptides were secreted extracellularly into the liquid media. Cell-associated bacteriocins produced by lactic acid bacteria had been reported previously [44], [45]. Hence, this class of bacteriocins can be recovered from producer cells grown in liquid media.

Most lantibiotics appear to be regulated at the transcriptional level in a cell-density-dependent manner in various bacteria [46]. The mode of regulation for lantibiotic production has been shown to involve secreted peptides that act as communication molecules accumulated in the environment during growth. When certain concentrations of these molecules are reached, high level of lantibiotic production is triggered [46]. A previous study demonstrated that the lantibiotic produced by strain NU10 is auto-regulated and the same lantibiotic could induce its production by strain NU10 [38]. However, strain NU10 was also shown to encode structural genes for salivaricins A and 9. But it was obvious that when an enhanced culture of strain NU10 was analysed using MALDI-TOF MS, salivaricin 9 was the only lantibiotic detected from the purified supernatant. Hence, we can conclude that the presence of structural genes encoding production of salivaricins in *S. salivarius* strains does not necessarily mean that the bioactive molecule is expressed or that the PTNYSMES medium used for the production in aerobic condition did not support the biosynthesis of that particular peptide.

Strain YU10 was shown to produce salG32 while no salivaricin A, 9 or streptin production was detected. Previous work showed that in contrast to the regulation of sal9, the signal of up-regulation of salivaricin G32 is not the antimicrobial peptide itself but rather some other substances produced by the lantibiotic producer [20].

The variety of bacteriocins produced by *S. salivarius* isolated from Malaysian subjects makes it interesting to study these molecules and their distribution among Malaysian population. High throughput genome sequencing of both strains NU10 and YU10 using Illumina’s MiSeq genome sequencing confirmed the absence of the streptococcal virulence determinants within both genomes (Figure 1) (Table 4). This finding nominates some of these strains as potentials for probiotic development as they pass the initial safety assessments described previously for *S. salivarius* strain K12 [39].

Bacteriocins and lantibiotics were not the only unique and useful molecules being produced by the strains described in this study. When sucrose was added to the medium as the only source of carbon, levan-sucrase enzyme was produced in significant levels. Levan-sucrase (fructosyltransferase) is a very unique cell-bound enzyme produced by *S. salivarius* and it plays an important role in the production of levan residues. Levan has been shown to have prebiotic effects and so this production, together with the production of lantibiotics, makes the strain potentially useful for multiple applications. The method described in this study for direct detection of levan-sucrase from the cell-extract using LC-MS/MS was efficient to detect levan-sucrase in *S. salivarius* and the full characterization of the gene encoding levan-sucrase production was elucidated using genome sequencing of the producer strain.

In conclusion, *S. salivarius* strains evaluated in this study showed critical variations in the type of inhibitory substances produced some of which are lantibiotics sal9 and salG32 produced by strains NU10 and YU10 respectively while gene encoding large bacteriocin molecule salMPS was detected in strain GT2. No significant variations in antibiotic susceptibility among *S. salivarius* isolates were observed after two years of storage indicating stability of the strains in terms of susceptibility towards antibiotics. The metabolic profile studies showed some variations among the tested strains and gave important information on the biochemical criteria required by each strain to perform better during fermentation studies. The *in vitro* safety assessment tests showed that the strains are free of virulence genes known to be present in streptococcal pathogens and this finding was supported by genome sequencing of strains NU10 and YU10.

Strains NU10 and YU10 produce sal9 and salG32 lantibiotics respectively which distinguish from the well characterised *S. salivarius* probiotic strain K12 producing the lantibiotics salA and salB. These differences introduce additional options for probiotics that may be used in oral health management with different lantibiotic molecules.

The developed medium PTNYSMES helped to enhance biomass accumulation of all strains and attempts to recover lantibiotics produced by *S. salivarius* grown in this medium aerobically were successful. A new method for levan-sucrase detection was also developed and gene encoding levan-sucrase production was characterized. The ability of *S. salivarius* to produce lantibiotics and levan-sucrase adds value to this microorganism with dual benefits for probiotic development with prebiotic effects.

## Materials and Methods

### 1. Bacterial Strains and Culture Media

*S. salivarius* strains NU10, YU10 and GT2 were isolated from the oral cavity of healthy Malaysian subjects and were deposited in the NCBI gene bank under accession numbers KC796011, KC796012 and KC796010 respectively. *S. salivarius* strain K12 was kindly provided by John Tagg (University of Otago, BLIS Technologies, New Zealand). Indicator strains including *Bacillus cereus* ATCC14579, *Lactococcus lactis* ATCC11454, *Micrococcus luteus* ATCC10240, *Streptococcus dysgalactiae* subsp. *equisimilis* ATCC12388, *Streptococcus pyogenes* ATCC12344, *Streptococcus pyogenes* ATCC12348, *Streptococcus sanguinis* ATCC10556 were purchased from American Type Culture Collection (ATCC). *Listeria monocytogenes* NCTC10890 was purchased from National Collection of Type Cultures. Other indicator strains such as *Actinomyces naeslundii* TG2, *Corynebacterium spp* GH17, *Enterococcus faecium* C1, *Haemophilus parainfluenza* TONEJ11, *Lactobacillus bulgaricus* M8, *Staphylococcus aureus* RF122, *Streptococcus gordonii* ST2, *Streptococcus mutans* GEJ11, *Weissella confusa* A3 were taken from the culture collection of Microbial Biotechnology Laboratory, Division of Microbiology, Institute of Biological Science, Faculty of Science, University of Malaya, Kuala Lumpur, Malaysia. Todd Hewitt broth (THB) (Difco) was used to propagate all the bacterial strains in this study. Mitis Salivarius agar (MSA) (Difco) was used to isolate pure colonies of *S. salivarius* strains. M17 (Merck), MRS (Merck), Brain Heart Infusion (BHI) (Difco) media were used to study the growth kinetics of *S. salivarius* strains. Columbia blood agar base (Difco) supplemented with 5% defibrinated sheep blood and 0.1% CaCO_3_ (BACa) was used to carry out the deferred antagonism test.

### 2. Ethical Approval for *S. salivarius* Sampling

The subjects were required to sign a consent form to isolate *S. salivarius* from the tongue surface using sterile cotton swab. Approval for sampling from tongue surface is not required from the IRB as a proforma for written consent from the subject is approved by the IRB. The ethics committee IRB Reference Number is DF OP1304/0019 (P) for our Institution (University of Malaya). This was discussed with the ethics committee (IRB) and the protocol used complied with Good Laboratory Practices. Therefore IRB approval is not required prior to sampling in this investigation.

### 3. Deferred Antagonism Test

The method was first described by [47] and performed in this study with some modifications. Bacteriocin producer strains were streaked across different production media agar plates as 1 cm wide strip using sterilized cotton swabs. The producers were then incubated aerobically with 5% CO_2_ for 18 hours at 37°C. Sterile cotton swabs were used to remove the bacteriocin producer bacteria before the plates were sterilized by inverting the plates over filter paper soaked with chloroform for 30 minutes. The plates were aired for another 30 minutes to get rid of any chloroform residues. Indicator bacterial strains of OD_600_ = 0.1 were streaked at a right angle across the producer streak. The plates were re-incubated under the same conditions mentioned above for 18 hours. Zones of no bacterial growth were recorded as antagonism activity due to bacteriocin production (Table 1). The antagonism assay for each strain was repeated twice using different production media.

### 4. DNA Extraction and Distribution of Genes Encoding Salivaricins Production

Single *S. salivarius* colonies grown on MSA medium for 18 hours were transferred to 10 ml of sterilized THB and incubated aerobically with 5% CO_2_ for 18 hours at 37°C before the pellets were centrifuged at 8000×*g* for 5 min at 4°C and suspended in 400 µl of 0.85% NaCl in water (w/v). The suspension was heated at 70°C for 30 min and the bacterial pellets were collected at 8000×*g* for 5 min at 4°C. The pellets were suspended in lysing buffer (2 mM Tris-HCl pH 8, 0.2 mM EDTA, 1.5% Triton X100 and 1000 U of mutanolysin) and incubated at 37°C for 2 hours. 50 µg/ml of lysozyme was added and the samples were further incubated for one hour at 37°C. After three freeze-thaw cycles, the samples were then processed using DNeasy Blood and Tissue kit (Qiagen) following manufacturer’s instructions for DNA extraction from Gram-Positive bacteria. PCR conditions for salivaricin genes amplifications were applied as described previously [16], [17], [20], [48] with some modifications to the reaction composition which included using Top Taq Master Mix (QIAGEN). This experiment was performed using Applied Biosystems Veriti 96-Well Thermo Cycler.

### 5. Biochemical Characterization of *S. salivarius* Isolates

All the biochemical tests were performed using 50 CH and 20 strep API kits to study the metabolic profiles of *S. salivarius* strains as shown in Table 2. The API kits were used according to manufacturer’s instructions (API-bioMeriex).

### 6. Antibiotics Susceptibility Test

The antibiograms of the three strains NU10, YU10 and GT2 were tested by the antibiotic disc diffusion assay according to Clinical and Laboratory Standards Institute (CLSI) (Vol. 32, No.3, Jan, 2012). *S. salivarius* cultures were grown on Mueller Hinton Agar (Difco, USA) supplemented with 5% sheep blood (Liofilchem srl, Italy) for 20 hours at 37°C in 5% CO_2_ atmosphere using BD Gas Pak EZ CO_2_ container system. Bacterial suspensions were prepared from morphologically identical colonies grown on the agar plates and suspended in saline solution (0.85% NaCl in water). The resultant bacterial suspensions were adjusted to turbidity of 0.5 McFarland before bacterial lawns were performed on the same blood agar plates mentioned above. Antimicrobial susceptibility test discs (OXOID, UK) were placed on top of the pre seeded plates using sterile forceps and the plates were incubated as mentioned above. Antibiotics used in this test are penicillin G, penicillin V, amoxicillin, ofloxacin, tetracycline, erythromycin, gentamicin, clindamycin, streptomycin, vancomycin, novobiocin and chloramphenicol. Both original cultures stored at −80°C from two years and weekly subcultures used in the lab routinely were tested to check for any differences following storage. Strain K12 was also tested as a control since it was reported previously [39] for susceptibility against the same and additional antibiotics used in this study. Measurement of the diameters of zones of complete inhibition (as judged by the unaided eye) including the diameter of the disc are listed in Table 3. This experiment was repeated in triplicates and showed almost identical results.

### 7. Preparation of the First Genome Drafts for Strains NU10 and YU10

Genome sequencing was carried out using Illumina’s compact MiSeq system at the High Impact Research Center, University of Malaya, Malaysia. Genomic libraries were prepared using the Nextera kit Illumina (Illumina, Inc., San Diego, CA) which produced a mean insert size between 800 and 1,200 bp. Total of 379-fold and 204-fold coverages were generated for strains NU10 and YU10 respectively. Approximately 85% of these reads were assembled using CLC Bio Genomic Workbench Software Version 6.0.5. Genome annotation was performed using RAST Version 4.0 [40]. The genome analysis included the virulence assessment for YU10 and NU10 strains to prove the absence of any streptococcal virulence determinants within both strains genomes (Table 4).

### 8. Developing New Bacteriocin-production Medium

Different media e.g. M17, MRS, THB and BHI were used to study the growth kinetics of *S. salivarius* grown aerobically at 37°C. Each *S. salivarius* strain was grown on BACa plates (Columbia agar base supplemented with 5% whole human blood and 0.1% CaCO_3_ for 18 hours at 37°C. Then the bacteria was washed from the agar plates using phosphate buffer saline at pH 7 and centrifuged to pellet the cells at 5000×*g* for 10 minutes. Bacterial pellets were washed twice with the same buffer using the same conditions mentioned above before re-suspending in the same buffer. The bacterial suspension was then diluted using the same buffer to 0.5 McFarland before 20 µl of each bacterial suspension was used to inoculate 180 µl of each medium into a 96-well sterile plate. The plate used in this experiment was flat base well plate and covered with a sterile plastic lid to prevent contamination. The growth was monitored by measuring the OD at a wavelength of 600 nm using a Multiskan GO Microplate Spectrophotometer (Thermo Scientific) over 24 hours. The spectrophotometer was set at medium speed shaking for 20 seconds before each photometric measurement. This experiment was carried out in triplicates and average of each triplicate measurement was used for growth kinetics (Figure 2). YNS medium (1% yeast extract, 1% neopeptone and 1% sucrose) and PTNYMES medium (1% peptone, 1% tryptone, 1% neopeptone, 1% yeast extract, 1% sucrose, 1% MES (2-(N-morpholino)ethanesulfonic acid), 0.2 g/L NaCl, 0.5 g/L ascorbic acid, 0.25 g/L magnesium sulphate and 0.2 g/L sodium acetate) were also used in this study. Typical compositions of all media used in this study are listed in Table 5.

### 9. Lantibiotics Production and Purification

PTNYMES medium (adjusted before autoclaving at pH 6.5 using concentrated NaOH) was inoculated with 5% of *S. salivarius* cultures grown for 18 hours in the same medium. 4000 mL shaking flasks were used for this experiment at 37°C for 22 hours with 150 rpm orbital shaking aerobically. The cultures were adjusted to pH 5.8 and incubated for 1 hour at 4°C to adsorb levels of lantibiotics secreted into the liquid medium to the producer’s cells. Then the cultures were centrifuged at 8500×*g* for 30 minutes and the cells were re-suspended in 95% methanol (adjusted to pH 2 by concentrated HCl). The cell suspensions were stirred gently overnight at 4°C for 18 hours before the cells were collected by centrifugation at 1000×*g* for 30 minutes. The supernatant was evaporated using a rotary evaporator at 45°C and the crude lantibiotic was assayed for antimicrobial activity. The crude preparation was concentrated 10 fold and then diluted 1∶5 (v/v) with 20 mM sodium phosphate buffer pH 5.8. This final preparation was subjected to FPLC ÄKTA purifier (GE Healthcare) using HiTrap SP FF strong cation exchanger column pre-equilibrated with 20 mM sodium phosphate buffer pH 5.8 (buffer A). The column was washed with 10X column volume of buffer A before a leaner gradient of buffer B (1 M NaCl in buffer A) was applied. Eluted fractions were collected using auto collector and the separation was monitored using three different UV wave lengths (207, 214 and 280 nm). Active fractions from 5 FPLC runs were pooled and concentrated before subjecting to Chromolith SemiPrep RP-18e 100-10 mm column using Waters HPLC system with gradient of 20% to 50% acetonitrile in water (v/v). UV wavelength of 214 nm was used to detect peptide peaks and the active fraction was subjected for the second time using the same column and conditions mentioned above to obtain the pure peptide. Well diffusion assay was performed to identify biologically active fractions using *Micrococcus luteus* ATCC10240 and *Streptococcus pyogenes* ATCC12344 as indicator targets. Pure lantibiotics were subjected to matrix assisted laser desorption ionization time of flight mass spectrometry MALDI-TOF (MS) using 4800 *Plus* MALDI TOF/TOF Analyzer to determine the molecular weight of the lantibiotic (Figure 3).

### 10. Production of Levansucrase

Strain YU10 was grown aerobically with 5% CO_2_ for 18 hours at 37°C in one litre of M17 medium supplemented with 2% yeast extract, 2% sucrose and 0.1% CaCO_3_ (M17YESUCa). The cells were collected by centrifugation at 18000×*g* for 5 min and re-suspended in 200 ml of 95% methanol adjusted to pH = 2 using concentrated HCl and incubated at 4°C for 18 hours before the supernatant was collected by centrifugation at 18000×*g* for 20 min. The methanol was evaporated using a rotary evaporator and the crude extract was lyophilized and kept at −20°C for further LC-MS/MS analysis.

### 11. Detection of Levan-sucrase by LC/MS-MS

The lyophilized extract was re-hydrated using 500 µl of 0.1% formic acid and injected into Water Oasis HLB column equilibrated with 0.1% formic acid. The sample was eluted with 600 µl 50% acetonitrile in 0.1% formic acid. The eluted sample was separated by reversed phase chromatography using a Thermo Scientific EASY-nLC II system with a reversed phase pre-column Magic C18 AQ (100 µm I.D., 2 cm length, 5 µm, 100 Å. Michrom Bio Resources Inc, Auburn, CA) attached to nano-analytical column Magic C18 (75 µm I.D., 15 cm length, 5 µm, 100 Å. Michrom Bio Resources Inc, Auburn, CA). The flow rate was set at 300 µl/min. The system was coupled to an LTQ OrbitrapVelos mass spectrometer equipped with a Nanospray II source (Thermo Fisher Scientific). Mobile phases were A (2% acetonitrile in 0.1% formic acid) and B (90% acetonitrile in 0.1 formic acid). After a 249 bar (∼5 µl) pre-column equilibration and 249 bar (∼8 µl) nano-column equilibration, the sample was separated by 55 min gradient as follows: (5% solvent B: 0 min, 40% solvent B: 60 min, 80% solvent B: 2 min and 80% solvent B: 8 min). The LTQ OrbitrapVelos (Thermo Fisher Scientific, Bremen, Germany) parameters were as follows: Nano-electrospray ion source with spray voltage 2.2 kV, capillary temperature 225°C, Survry MS1 scan *m/z* range from 400 to 2000 profile mode, resolution 60,000 at 400 *m/z* with AGC target 1E6 and one microscan with maximum inject time 200 ms. Lock mass Siloxane 445.120024 for internal calibration with preview mode for FTMS master scan: on, injection waveforms: on, monoisotopic precursor selection: on, rejection of charge state: 1. The sample was analysed with a top-5 most intense ions charge state 2–4 exceeding 5000 counts were selected for CID FT-MSMS fragmentation and detection in centroid mode. Dynamic exclusion setting were: repeat count 2, repeat duration 15 seconds, exclusion list size 500, exclusion duration 60 seconds with a 10 ppm mass window. The CID activation isolation window was: 2 Da, AGC target: 1E4, maximum inject time: 25 ms, activation time: 10 ms, activation Q: 0.250 and normalized collision energy 35%.

### 12. Data Analysis Parameters

Proteome Discoverer 1.3.0.339 software suite (Thermo Scientific) was used to analyze raw files. Parameters for the spectrum selection to generate peak lists of the CID spectra were as follows: activation type: CID, s/n cut-off: 1.5, total intensity threshold: 0, minimum peak count: 1, precursors mass: 350–5000 Da. The peak lists were submitted to an in-hose mascot 2.2 against the Uniprot-Swissprot databases (Figure 4).

## Supporting Information

Figure S1 Comparison of regions for genes encoding levan-sucrase (fructosyltransferase) enzyme in *S. salivarius* YU10 and *S. salivarius* M18.
(TIF)

Text S1 Lantibiotic peptides detected in *S. salivarius* YU10 genome using SEED viewer software version 4.0.
(DOCX)

Text S2 DNA to protein translation of levan-sucrase or fructosyltransferase (FTF) of *S. salivarius* YU10. Highlighted residues are those detected by ESI-LC-MS/MS.
(DOCX)
