# Supplementary figures and images for: Variable Characteristics of Bacteriocin-Producing Streptococcus salivarius Strains Isolated from Malaysian Subjects
Source: PLoS One. 2014 Jun 18;9(6):e100541. doi: 10.1371/journal.pone.0100541 (PMC4062538; doi:10.1371/journal.pone.0100541)

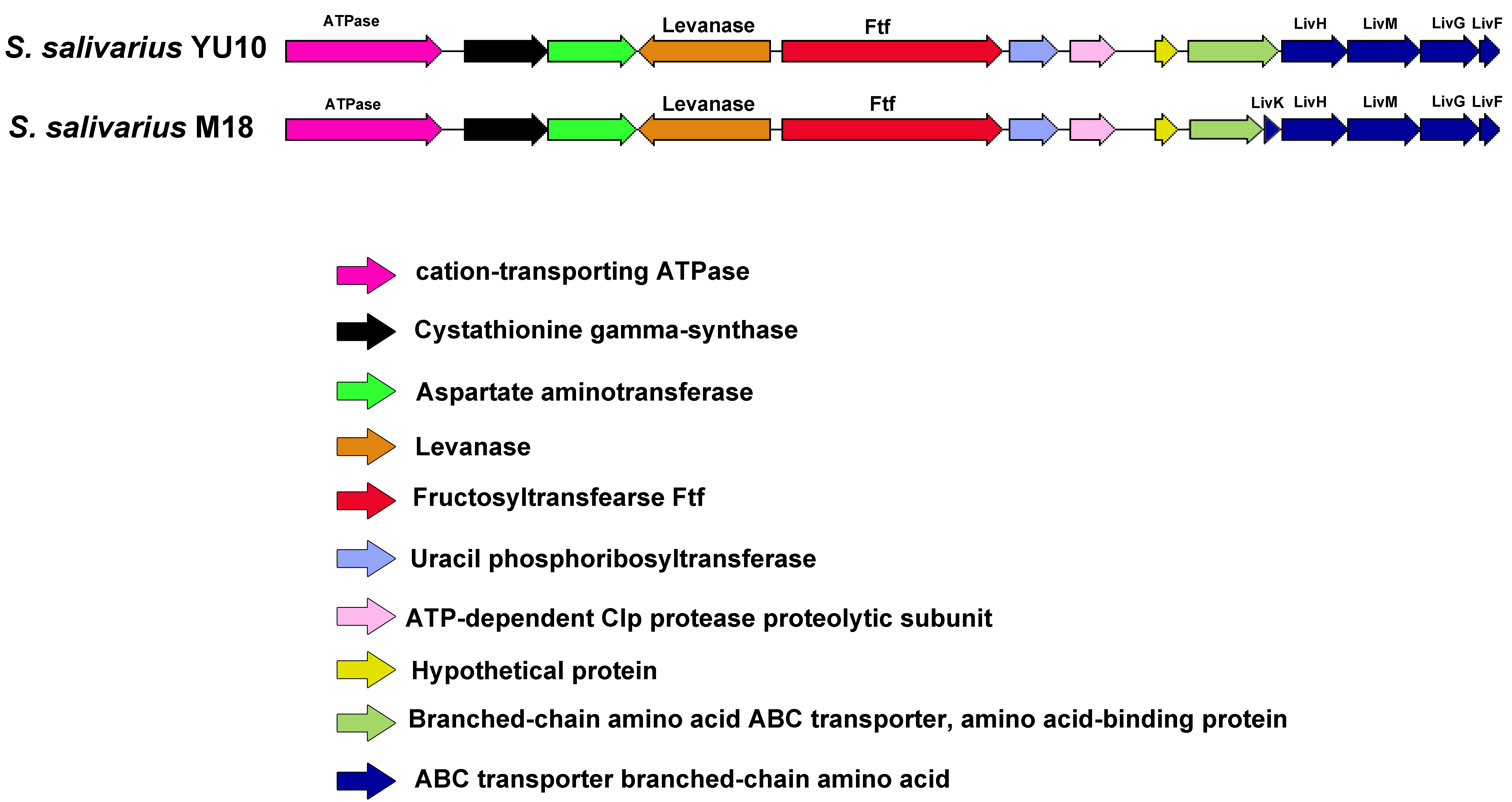

Supplement: Figure S1 — Comparison of regions for genes encoding levan-sucrase (fructosyltransferase) enzyme in S. salivarius YU10 and S. salivarius M18. (TIF) [file pone.0100541.s001.tif]
