# Supplementary material for: Variable Characteristics of Bacteriocin-Producing Streptococcus salivarius Strains Isolated from Malaysian Subjects
Source: PLoS One. 2014 Jun 18;9(6):e100541. doi: 10.1371/journal.pone.0100541 (PMC4062538; doi:10.1371/journal.pone.0100541)
Supplement: Text S1 — Lantibiotic peptides detected in S. salivarius YU10 genome using SEED viewer software version 4.0. (DOCX) [file pone.0100541.s002.docx]

**Text S1.** Lantibiotic peptides detected in *S. salivarius* YU10 genome using SEED viewer software version 4.0

**1. Streptin lantibiotic**

**>****[fig|6666666.65350.peg.1401](http://rast.nmpdr.org/seedviewer.cgi?page=Annotation&feature=fig%7C6666666.65350.peg.1401)**

**Length = 46**

**Score = 85.5 bits (210), Expect = 3e-19**

**Identities = 38/46 (82%), Positives = 40/46 (86%)**

**Query: 1 MNNTIKDFDLDLKTNKKDTATPYVGSRYLCTPGSCWKLVCFTTTVK 46**

**M NTIKDFDLDLKT K ++ P VGSRYLCTPGSCWKLVCFTTTVK**

**YU10 : 1 MKNTIKDFDLDLKTTKNNSDEPLVGSRYLCTPGSCWKLVCFTTTVK 46**

**2. Salivaricin A3 lantibiotic**

**>****[fig|6666666.65350.peg.1498](http://rast.nmpdr.org/seedviewer.cgi?page=Annotation&feature=fig%7C6666666.65350.peg.1498)**

**Length = 48**

**Score = 103 bits (258), Expect = 8e-25**

**Identities = 48/48 (100%), Positives = 48/48 (100%)**

**Query: 1 MKNSKDVLNNAIEEVSEKELMEVAGGKKGPGWIATITDDCPNSIFVCC 48**

**MKNSKDVLNNAIEEVSEKELMEVAGGKKGPGWIATITDDCPNSIFVCC**

**YU10 : 1 MKNSKDVLNNAIEEVSEKELMEVAGGKKGPGWIATITDDCPNSIFVCC 48**

**3. Salivaricin G32 lantibiotic**

**>****[fig|6666666.65350.peg.1506](http://rast.nmpdr.org/seedviewer.cgi?page=Annotation&feature=fig%7C6666666.65350.peg.1506)**

**Length = 49**

**Score = 103 bits (258), Expect = 8e-25**

**Identities = 49/49 (100%), Positives = 49/49 (100%)**

**Query: 1 MKKDVVIESIKEVSLEELDQIIGAGNGVFKTISHECHLNTWAFLATCCS 49**

**MKKDVVIESIKEVSLEELDQIIGAGNGVFKTISHECHLNTWAFLATCCS**

**YU10 : 1 MKKDVVIESIKEVSLEELDQIIGAGNGVFKTISHECHLNTWAFLATCCS 49**

**4. SlnA1 putative lantibiotic-like protein**

**>****[fig|6666666.65350.peg.1504](http://rast.nmpdr.org/seedviewer.cgi?page=Annotation&feature=fig%7C6666666.65350.peg.1504)**

**Length = 52**

**Score = 110 bits (276), Expect = 7e-27**

**Identities = 52/52 (100%), Positives = 52/52 (100%)**

**Query: 1 MKNNKIHLEALEALQELKMEEIDNLLGGAGHGVNTISAECRWNSLQAIFTCC 52**

**MKNNKIHLEALEALQELKMEEIDNLLGGAGHGVNTISAECRWNSLQAIFTCC**

**YU10 : 1 MKNNKIHLEALEALQELKMEEIDNLLGGAGHGVNTISAECRWNSLQAIFTCC 52**
