# Supplementary material for: Variable Characteristics of Bacteriocin-Producing Streptococcus salivarius Strains Isolated from Malaysian Subjects
Source: PLoS One. 2014 Jun 18;9(6):e100541. doi: 10.1371/journal.pone.0100541 (PMC4062538; doi:10.1371/journal.pone.0100541)
Supplement: Text S2 — DNA to protein translation of levan-sucrase or fructosyltransferase (FTF) of S. salivarius YU10. Highlighted residues are those detected by ESI-LC-MS/MS. (DOCX) [file pone.0100541.s003.docx]

**Text S2**. DNA to protein translation of Levan-sucrase or fructosyltransferase (FTF) of *S. salivarius* YU10.

>ORF sequence | 971 aa

MDSTVNSQSNTVAPKQAECKKMRYSIRKVATVGATSALVGTLAFLGATQVKADQVTETAP 60

AVATATATPETSTASLTVASEAATSVATSEAVESSVAHSEVATTPVTETQPSNTTPSVVE 120

EKVSSTVVTSSSDATTPSATVAAVSAPAHTSEAAVEAPTSTASSETADTHTEVALKPTEN 180

SAANANLSKLNGRIKSIVEDNMTSDQIVALTEEEIKALNKVDFSDDAIKGTGTSLTYRNL 240

KDIVASFLKQDSKLAVPYFKADTIINMPAFNTVDAQTMKKEEIDVWDSWPVQDAESGVVS 300

NWNGYQLVISMAGAPNKNSNHIYLLYSKYGDNDFTHWKNAGPIFGYNALEDDQQWSGSAT 360

VNSDGSIQLYYTKNDTSGGKLNWQQLASATLNLAVENDEVVIKSVENDHILFGGDNYHYQ 420

SYPKFMSTFNDDHNHDGNPDRTDNYCLRDPHIIEDNGSRYLIFESNTGDENYQGEKQIYN 480

WSNYGGDDAFNLKSFLNIVNNKHLYNLASWANGSIGILKLDDNEKNPSVAELYTPLVTSH 540

MVTDEVERPSVVKMGNKYYLFTASRINKSTDAEGTVAAREAVGDDVVMLGFVSDSLRGEY 600

RPLNGSGVVLTASVPADWRTSTYSYYAVPVEGSSDTLLVTSYMTNRGGIAGAENKSTWAP 660

SFLIKMNEDDTTEVLPKMTNQGDWIWDKSSESLVHVADQNSAKLPNEDYNVDYYAVSDYG 720

LKPHTYPTVDGSPGVSEARGVLTVTVKDGEDKKSDKSETPVNPTEGNHSVDDKSNKPDTP 780

SKPADNNQPSTNKEDKPATPTNPDSPVRNPFPYFTDRPSNDNNSSDDHHVEVPAKPSTES 840

SVGDRRPVAQATEIASPVPEAIVATGPTVSTTPVKEESVTETEAPKPAKSEEEVQSHGVA 900

KADEVTKSDESSKDNNTKVAAKLATTPKTPSDSEGSKSNILSILATIFAAIASLALLGYG 960

LVTGKIHLPKK 971

Highlighted residues are those detected by ESI-LC-MS/MS

DNA sequencing of gene encoding Levan-sucrase or fructosyltransferase (FTF) of *S. salivarius* YU10

>DNA seq 2913

ATGGATAGTA CAGTTAATTC ACAGTCAAAT ACAGTAGCAC CTAAGCAAGC AGAGTGCAAA 60

AAGATGCGTT ATAGCATCCG TAAGGTAGCC ACAGTAGGGG CAACATCAGC ACTCGTTGGT 120

ACCTTGGCAT TTTTGGGTGC CACTCAGGTA AAAGCTGATC AAGTTACAGA AACAGCACCA 180

GCTGTAGCTA CTGCGACAGC AACACCAGAA ACAAGCACAG CATCTCTAAC GGTAGCCAGT 240

GAAGCAGCAA CAAGTGTGGC AACTTCAGAA GCAGTTGAAT CTTCAGTTGC ACATTCAGAG 300

GTTGCAACTA CACCAGTTAC AGAGACACAA CCAAGCAACA CAACTCCATC AGTAGTCGAA 360

GAAAAGGTGT CTAGCACAGT AGTGACATCT TCATCAGATG CAACAACACC TTCAGCAACT 420

GTTGCTGCGG TTTCTGCTCC AGCTCATACA TCAGAGGCTG CAGTAGAGGC ACCAACTTCA 480

ACAGCAAGCT CAGAAACAGC TGATACACAC ACTGAAGTAG CCCTTAAACC TACTGAAAAC 540

AGCGCTGCTA ATGCAAACCT TAGCAAACTT AACGGTCGTA TCAAGTCTAT TGTTGAAGAT 600

AATATGACCT CAGATCAAAT CGTTGCCTTG ACTGAGGAAG AAATTAAAGC ACTTAATAAA 660

GTTGACTTTA GCGATGATGC TATCAAGGGT ACAGGTACTA GCTTGACTTA CCGTAACTTG 720

AAAGATATCG TTGCTAGCTT CTTGAAGCAA GACAGCAAAT TGGCAGTTCC TTACTTTAAA 780

GCAGATACAA TCATCAATAT GCCTGCTTTC AACACTGTTG ATGCCCAAAC GATGAAAAAA 840

GAAGAAATCG ACGTTTGGGA TTCTTGGCCA GTCCAAGATG CTGAGTCAGG TGTAGTAAGC 900

AACTGGAACG GTTATCAGTT GGTTATCTCA ATGGCCGGTG CACCAAATAA AAACTCAAAC 960

CACATTTACT TGCTCTATAG TAAATACGGA GATAATGACT TTACACATTG GAAAAATGCG 1020

GGTCCAATCT TTGGTTATAA TGCCCTTGAA GATGACCAAC AATGGTCAGG TTCAGCGACG 1080

GTAAACTCTG ACGGTAGTAT CCAACTTTAC TACACTAAGA ATGATACTAG CGGCGGTAAA 1140

TTGAACTGGC AACAATTGGC TAGTGCTACA CTTAACCTAG CTGTTGAAAA CGACGAAGTT 1200

GTTATTAAGT CAGTTGAAAA TGACCACATT CTCTTTGGTG GGGATAACTA TCATTATCAA 1260

AGTTATCCAA AATTCATGAG TACTTTCAAC GATGACCACA ACCACGATGG AAATCCAGAC 1320

CGTACAGATA ACTACTGTCT TCGTGACCCA CACATCATTG AAGATAACGG TAGCCGTTAC 1380

CTTATTTTCG AATCAAATAC AGGTGATGAA AACTACCAAG GCGAAAAACA AATCTACAAC 1440

TGGTCAAACT ATGGAGGAGA TGACGCCTTT AACCTTAAGT CATTCCTCAA TATTGTCAAT 1500

AATAAACACC TTTATAACCT TGCTTCATGG GCAAATGGTT CTATTGGTAT CTTGAAACTT 1560

GATGACAATG AGAAAAATCC ATCTGTAGCT GAACTATACA CACCACTTGT TACTAGCCAC 1620

ATGGTTACTG ATGAAGTAGA ACGTCCAAGT GTTGTTAAGA TGGGTAACAA GTATTACCTC 1680

TTCACTGCTT CACGTATCAA TAAATCTACG GATGCTGAAG GTACAGTAGC AGCGCGTGAA 1740

GCTGTCGGTG ATGACGTTGT TATGCTTGGA TTCGTATCAG ATAGCCTCCG TGGAGAATAC 1800

CGTCCACTTA ATGGCTCAGG TGTGGTTTTG ACAGCCTCTG TACCAGCTGA CTGGCGTACA 1860

TCTACCTATT CTTACTATGC GGTTCCTGTT GAAGGATCAT CAGATACTCT TTTGGTAACT 1920

TCTTACATGA CTAACCGTGG TGGTATTGCT GGTGCTGAGA ATAAATCGAC ATGGGCTCCA 1980

AGCTTCCTCA TTAAGATGAA TGAAGATGAT ACTACAGAAG TTCTTCCTAA GATGACCAAC 2040

CAAGGGGACT GGATTTGGGA TAAATCTAGT GAAAGCTTGG TACACGTGGC CGATCAAAAC 2100

AGTGCTAAAT TGCCAAATGA GGACTATAAC GTAGATTATT ATGCAGTGTC AGATTATGGT 2160

CTTAAACCAC ATACTTATCC TACAGTTGAT GGTTCACCGG GCGTTTCTGA AGCTCGCGGT 2220

GTCTTAACTG TAACTGTTAA AGATGGTGAA GACAAGAAAT CAGATAAATC AGAAACACCA 2280

GTAAATCCAA CAGAAGGTAA TCATTCTGTT GATGATAAGT CTAACAAACC AGATACACCT 2340

TCTAAACCAG CAGACAATAA TCAACCATCA ACTAATAAAG AAGATAAACC AGCAACGCCG 2400

ACGAATCCAG ATTCTCCAGT TCGTAATCCG TTCCCTTATT TCACAGACCG CCCATCTAAT 2460

GATAATAATT CATCAGATGA TCATCATGTA GAGGTTCCTG CGAAACCTTC AACAGAGAGT 2520

TCTGTGGGGG ACCGTCGTCC AGTAGCTCAA GCTACAGAGA TTGCGTCACC AGTACCAGAG 2580

GCTATTGTAG CAACTGGACC AACTGTTTCA ACGACTCCTG TTAAAGAAGA GTCTGTCACT 2640

GAAACAGAAG CTCCAAAACC AGCTAAGTCT GAAGAGGAAG TTCAGTCTCA CGGAGTCGCT 2700

AAAGCTGATG AAGTAACTAA GTCTGATGAG TCAAGTAAAG ACAACAATAC TAAAGTTGCT 2760

GCCAAACTTG CTACGACTCC TAAGACACCA AGTGATTCAG AAGGTTCAAA GAGCAACATT 2820

CTTTCAATCT TGGCGACTAT CTTTGCAGCA ATTGCAAGTC TAGCTCTTCT TGGCTATGGT 2880

TTGGTCACAG GAAAGATTCA TTTGCCTAAA AAA
